# Heat acclimation mediates cellular protection via HSP70 stabilization of HIF-1α protein in extreme environments

**DOI:** 10.7150/ijbs.103122

**Published:** 2025-01-01

**Authors:** Can Li, Jirui Wen, Ling Wang, Jun Lei, Zhengdong Lin, Yuhao Zou, Juan Cheng, Xuehong Wan, Jifeng Liu, Jiang Wu

**Affiliations:** 1 Department of Otolaryngology Head and Neck Surgery/Deep Underground Space Medical Center, West China Hospital, Sichuan University, Chengdu, China.; 2 State Key Laboratory of Intelligent Construction and Healthy Operation and Maintenance of Deep Underground Engineering, Sichuan University, Chengdu, China.; 3 Med-X Center for Manufacturing, Sichuan University, Chengdu, China.; 4 Jinping Deep Underground Frontier Science and Dark Matter Key Laboratory of Sichuan Province, Liangshan, China.

**Keywords:** Heat acclimation, Heat stress, Hypoxia, Ubiquitination, Hypoxia-inducible factor 1-alpha, Heat shock protein 70

## Abstract

Heat acclimation (HA) is an evolutionarily conserved trait that enhances tolerance to novel stressors by inducing heat shock proteins (HSPs). However, the molecular mechanisms underlying this phenomenon remain elusive. In this study, we established a HA mouse model through intermittent heat stimulation. Subsequently, this model was evaluated using an array of physiological and histological assessments. *In vitro*, HA cell model with mouse brain microvascular endothelial cells (bEnd.3) was established and analyzed for cell viability and apoptosis markers. We investigated HA-mediated heat and hypoxia tolerance mechanisms using HIF-1α and HSP70 inhibitors and siRNA. Our results demonstrated that HA enhances the tolerance of bEnd.3 cells and mice to both heat and hypoxia, Mechanistically, HA upregulated the expression of HIF-1α and HSP70. However, inhibition of HIF-1α or HSP70 partially attenuated HA-induced tolerance to heat and hypoxia. Additionally, HA significantly decreased the ubiquitination levels of HIF-1α, whereas inhibition of HSP70 increased its ubiquitination. HA also substantially enhanced the interaction between HIF-1α and HSP70. In conclusion, our findings indicate that HA enhances tolerance to heat and hypoxia by stabilizing HIF-1α through increased interaction with HSP70. This discovery elucidates a novel mechanism of cellular protection conferred by HA and provides new strategies and potential targets for human adaptation to extreme environments.

## Introduction

Extreme environments on earth, such as the desert heat and high-altitude low oxygen, pose significant challenges to human survival 1, 2. Global climate change is increasing the frequency of extreme weather events, worsening these conditions. Studying human adaptation mechanisms to these environments deepens our understanding of adaptation physiology and may offer new strategies to cope with environmental changes, enhancing human survival in diverse conditions.

HA, a natural phenomenon prevalent in the biological world, has garnered significant attention from the scientific community. It refers to an organism's enhanced tolerance to heat stress (HS) through exposure to higher temperature environments 3, 4. Increasing evidence suggests that HA not only improves the tolerance of organisms to high temperature but may also enhance adaptability to hypoxic conditions through molecular and cellular mechanisms. This phenomenon of cross-tolerance implies that HA possibly induces the production of protective molecules, such as HSPs and HIFs, thereby increasing the resilience of organism to hypoxic stress 5, 6.

Hypoxia-inducible factor 1-alpha (HIF-1α) is a central transcription factor responsible for regulating the expression of a series of genes involved in erythropoiesis, glucose transport, glycolysis, and angiogenesis, thereby enhancing oxygen delivery and optimizing metabolism under hypoxic conditions 7. Heat shock protein 70 (HSP70) functions as a molecular chaperone, protecting cells from damage under various stress conditions by interacting with other proteins, aiding in their proper folding, preventing aggregation and degradation, and potentially influencing the function of HIF-1α directly or indirectly 8, 9. Notably, studies have demonstrated that HSP70 acts as a downstream effector of HIF-1α, contributing to the resistance against hypoxic damage in cells 10, 11. In tumor cells, HS induces the upregulation of HSP70 expression, which further promotes the post-translational modification of HIF-1α, exacerbating the malignant characteristics of tumor cells 12. Notably, HSP70 and HIF-1α may exert synergistic effects in cross-tolerance mediated by HA, helping cells to establish a "pre-conditioning state" after HA, which in turn facilitates a rapid response to new stressor stimuli 13. However, the specific molecular mechanisms of this process remain unclear. Therefore, exploring how HA mediates cross-talk between HSP70 and HIF-1a not only deepens our understanding of how organisms cope with multiple environmental stresses through intrinsic mechanisms but also may pave the way for more effective and safer extreme environments adaptation strategies.

In this study, we successfully established HA cellular and mouse models through intermittent heat stimulation. These models exhibited significant tolerance to both high temperature and hypoxic conditions. From a molecular mechanism perspective, we discovered that HA significantly increased the expression of HIF-1α and HSP70 proteins. Importantly, HA strengthened the interaction between these two proteins, inhibiting the degradation of HIF-1α via the ubiquitin-proteasome pathway. In summary, our research demonstrates that intermittent heat stimulation effectively induces HA, enhancing tolerance to high temperatures and hypoxia, and this effect may depend on the cross-talk of the HIF-1α/HSP70 signaling.

## Materials and Methods

### Reagent

BioTracker ERthermAC (SCT057, Merck, Darmstadt, Germany), HSP70 inhibitor (VER-155008, HY-10941, MCE, New Jersey, USA), HIF-1α inhibitor (LW6, HY-13671, MCE), Cycloheximide (CHX, HY-12320, MCE), Vari Fluor 488-Phalloidin (HY-D1817, MCE), Primary antibodies: anti-β-Actin (1:10000, 60008-1-Ig, Proteintech, Illinois, USA), anti-Ubiquitin (1:3000, 10201-2-AP, Proteintech), anti-P53 (1:3000, 60283-2-Ig, Proteintech), anti-Bcl2 (1:3000, 26593-1-AP, Proteintech), anti-Bax (1:2000, 60267-1-Ig, Proteintech), anti-Cleaved-Caspase3 (1:3000, 25128-1-AP, Proteintech), anti-HIF-1α (1:3000, #9542, CST, USA), anti-HSP70 (1:5000, CY5496, Abways, Shanghai, China), anti-HSP90 (1:5000, BM4387, BOSTER, California, USA), Hematoxylin & Eosin stain (HE, C0105S, Beijing, China), Nissl stain (C0117, Beyotime), Matrigel (Corning, 354234, New York, USA), Cell counting kit-8 (CCK-8, K1018, APExBIO Houston, USA), Dulbecco's Modified Eagle Medium-High glucose (DMEM, C11875500BT, Gbico, Waltham, USA), Fetal Bovine Serum (FBS, BS1612-109, Bioexplorer, Caesarea, Israel), Penicillin/streptomycin (P/S, BL505A, Biosharp, Beijing, China), ATP assay kit (S0026, Beyotime), TUNEL assay kit (C1088, Beyotime), BCA assay kit (P0010S, Beyotime), RIPA (P0038, Beyotime), Annexin V-FITC apoptosis detection kit (C1062M, Beyotime), Lipofectamine 3000 (L3000-008, Invitrogen, Waltham, USA), Evans blue (EB, E2129, Sigma-Aldrich, Missouri, USA).

### Cell culture and transfection

The bEnd.3 cell line was purchased from Procell Life Science & Technology Co., Ltd. (Wuhan, China). The bEnd.3 cells were cultured in DMEM-high glucose culture medium, supplemented with 10% FBS and 1% P/S. The cells were maintained in an incubator at 37°C with an atmosphere containing 5% CO_2_. The complete culture medium was refreshed for every three days.

In bEnd.3 cells, siRNA was used to knock down *Hif-1α* and *Hspa1a* mRNA (RiboBio, Guangzhou, China). The *Hspa1a* siRNA pool contains three siRNAs: siRNA-56 (siRNA *Hspa1a*-1), siRNA-1274 (siRNA *Hspa1a*-2), and siRNA-1688 (siRNA *Hspa1a*-3). The siRNA sequences are listed in Table S1; si-*NC* was used as the negative control.

### Establishment of HA cells

bEnd.3 cells were passaged and seeded into 6 cm culture dishes, then incubated at 37℃ with 5% CO_2_. The next day, the cells were exposed to 40℃ incubator with 5% CO_2_ for 2 h, then transferred back to 37℃. The process was repeated for 1, 3, and 5 days as described.

### Cell viability and ATP assay

The control cells (bEnd.3) and HA cells (bEnd.3^HA1d^, bEnd.3^HA3d^, bEnd.3^HA5d^) were digested, centrifuged, and resuspended. The cells were then seeded into a 96-well plate at a density of 1×10^^3^ cells per well and exposed to 43°C incubator with 5% CO_2_ for 0, 2, 4, and 6 h, respectively. Afterward, CCK-8 solution was added, and the plates were incubated at 37°C for 1 h. Absorbance was measured at 450 nm using a microplate reader.

Following the same treatment, ATP levels were measured using a multifunctional microplate reader (SuPerMax 2800MF, Shanghai Flash Spectrum Biotechnology Co., Ltd., Shanghai, China) following the manufacturer's instructions.

### Cell apoptosis assay

The bEnd.3, bEnd.3^HA^ cells, and bEnd.3^HA^ cells transfected with si-NC, si-*Hif-1α*, si-*Hspa1a*, or si-*Hif-1α* + si-*Hspa1a* were exposed to 43°C incubator with 5% CO_2_ for 4 h. Cells were then collected and processed according to the reagent instructions. Annexin V-FITC and PI reagents were added, and samples were incubated in the dark at room temperature for 15 min. Apoptosis rates were detected using a flow cytometer.

After the aforementioned groups of cells were cultured in a 37°C incubator with 1% O_2_ for 12 h, and apoptosis detection was performed as previously described.

### IF staining

The bEnd.3 and bEnd.3^HA^ were digested, centrifuged, and resuspended, then seeded into a 24-well plate at a density of 2×10^^4^ cells per well. The plates were exposed to 43°C incubator with 5% CO_2_ for 4 h. After incubation, cells were fixed with 10% neutral formaldehyde, permeabilized with 0.3% Triton X-100, and blocked with 10% goat serum. Primary antibodies (anti-HIF-1α, anti-HSP70) were added and incubated overnight at 4°C in the dark. Cells were then washed with PBS, incubated with fluorescent secondary antibodies for 2 h at 37°C, stained with DAPI, mounted with glycerol, and observed under a fluorescence microscope (Nikon, Tokyo, Japan).

Brain microvessels from different mouse groups were fixed with 10% neutral formaldehyde, air-dried on slides, and permeabilized with 0.3% Triton X-100 for 10 min. After washing with PBS, Tunel staining solution was added and incubated at 37°C in the dark for 60 min. The samples were then washed with PBS, mounted with glycerol, and observed under a fluorescence microscope.

### Angiogenesis experiment

A 24-well cell culture plate was placed on ice, and 0.289 mL of cold Matrigel (10 mg/mL) was pre-added to each well. The plate was then incubated at 37°C for 30 min. Subsequently, 300 µL of cell suspension (1.2 × 10^^5^ cells per well) from different treatment groups was added to each well. The plate was further incubated at 37°C with 5% CO_2_ for 4 h.

### Western blot (WB) analysis

Tissues and cells were lysed using RIPA buffer to extract total protein. Protein concentrations were determined with a BCA protein assay kit. Proteins were separated by 10% SDS-PAGE and transferred to a PVDF membrane. After blocking and washing, the membrane was incubated with specific primary antibodies at 4°C overnight. Following three washes, the membrane was incubated with a secondary antibody. The density of the blot bands was quantified using ImageJ software (National Institutes of Health, Bethesda, MD). Protein expression levels are normalized to β-actin.

### Molecular docking analysis

Rigid protein-protein docking (ZDOCK) was performed between HSP70 and HIF-1α to study the relationships. The PDB format of the protein structural domain wa downloaded from the protein Data Bank PDB database (http://www.rcsb.org/). The ZDOCK module wa run to identify the docking sites and calculate the ZDOCK scores.

### Co-immunoprecipitation (CO-IP) assay

Cell lysates were collected, and 2 μg of anti-HIF-1α or HSP70 antibodies were added, with homologous IgG antibodies used as controls. The mixture was incubated at 4°C overnight. Protein A/G was then added and mixed at 4°C for 3 h. After washing and centrifugation, the pellet was resuspended in 30 μL of sample buffer. Following further centrifugation, the supernatant was collected to obtain immune complexes. As described previously, the immune complexes were separated by WB and transferred to a PVDF membrane. Finally, immunoblotting was performed using anti-HIF-1α, HSP70, and Ubiquitin antibodies according to the manufacturer's specified dilutions.

### Real-time qPCR

All gene primer sequences were synthesized by Tsingke Biological Technology Company (Beijing, China). Total RNA extraction, cDNA synthesis, and qPCR were conducted as described previously 14. In addition, mRNA was reverse transcribed using RT Easy^TM^ II kit (With gDNase; 1.0-1904, Foregene, Chengdu, China), and qPCR was performed using Real Time PCR Easy^TM^-SYBR Green I kit (1.0-1403; Foregene). Primer sequences for RT-qPCR are listed in Table S2.

### HA and hypobaric hypoxia in mice

Healthy male C57/BJ mice (n = 24), weighing 25 ± 2 g, were randomly divided into three groups: Control, Hypoxia, and Hyp + HA group. The HA process for the mice was established as follows: the mice were placed in a high-temperature chamber (40°C, 40-50% humidity; (20-DX-4-001, Shandong, China) for 30 min daily for 10 days. Immediately following, the Hypoxia and Hyp + HA group mice were placed in a hypobaric chamber (20-DX-4-001, Shandong, China) for 12 h at 40-50% humidity and 23-25°C. This setup allowed for the analysis of changes in blood-brain barrier permeability (BBB), blood routine, biochemistry, and histopathology induced by hypoxia. According to previous reports 15, 16, an altitude of 8,300 meters (7.0% O_2_) was chosen to create hypobaric hypoxia conditions. Under these conditions, typical hypoxic organ damage sufficient to allow survival was induced in mice.

All animal operations were conducted in line with the Chinese Guidelines for Use of Experimental Animals, which was also approved by the Animal Experimentation Ethics Committee, the West China Hospital of Sichuan University (No.20240304101).

### HA and HS in mice

Healthy male C57/BJ mice (n = 24), weighing 25 ± 2 g, were randomly divided into three groups: Control, HS, and HS + HA group. HA treatment was applied as previously described. The HS and HS + HA groups mice were placed in a high-temperature (39°C) chamber with 40-50% humidity for 2.5 h 17. After HS exposure, the mice were returned to their original cages at 25°C with free access to food and water. Control animals underwent the same procedure without HS exposure. This setup enabled the analysis of serum inflammatory cytokines, histopathology, and protein levels induced by high temperature.

### BBB permeability test

Evans Blue (EB) was dissolved in physiological saline to a 2% concentration and administered to mice via the tail vein at 5 mL/kg before hypoxia experiments. After 12 h, the mice were perfused with physiological saline through the left ventricle until the effluent from the right atrium was colorless, removing blood and unbound dye from the brain. Brain tissues were then harvested, weighed, and homogenized in methanol. The homogenate was centrifuged, and the supernatant containing EB was collected. The absorbance of EB in the supernatant was measured at 610 nm, and its concentration in the brain tissue was quantified using a standard curve, expressed as mg of EB per gram of brain tissue.

### Isolation of mouse brain microvessels

Whole brains were excised from each group of mice. After removing the cerebellum and olfactory bulbs, the remaining brain tissue was rapidly immersed in liquid nitrogen and subsequently stored at -80°C. The brain tissue was homogenized in 3 mL of cold sucrose buffer (0.32M sucrose, 5mM HEPES, pH 7.4). The resulting homogenate was centrifuged at 1000 × g for 10 min, and the supernatant, containing most neural components, was discarded along with the dense myelin layer atop the pellet. The pellet was resuspended in 3 mL of sucrose buffer and centrifuged again at 1000×g for 10 min to remove residual myelin. Subsequently, low-speed centrifugation at 40 × g was employed to separate large blood vessels from capillaries. The pellet was washed four times with 1 mL of sucrose buffer, each wash followed by centrifugation at 350 × g for 10 min. A portion of the pellet was preserved on a slide and stored at -80°C for future analysis, while the remaining portion was used for total protein extraction.

### Statistical analysis

All statistical analyses were conducted using GraphPad Prism 9. Data are presented as mean ± SD. Two groups were compared using unpaired two-tailed Student's *t*-test, and more than two groups were compared using a one-way analysis of variance (ANOVA), with significant omnibus tests further interrogated through post-hoc Tukey test. A *P*-value of less than 0.05 was considered statistically significant (*P < 0.05, **P < 0.01, ***P < 0.001, ****P < 0.0001). All experiments were repeated at least three times.

## Results

### Intermittent heat stimulation enhances cellular thermotolerance

**Figure 1:**
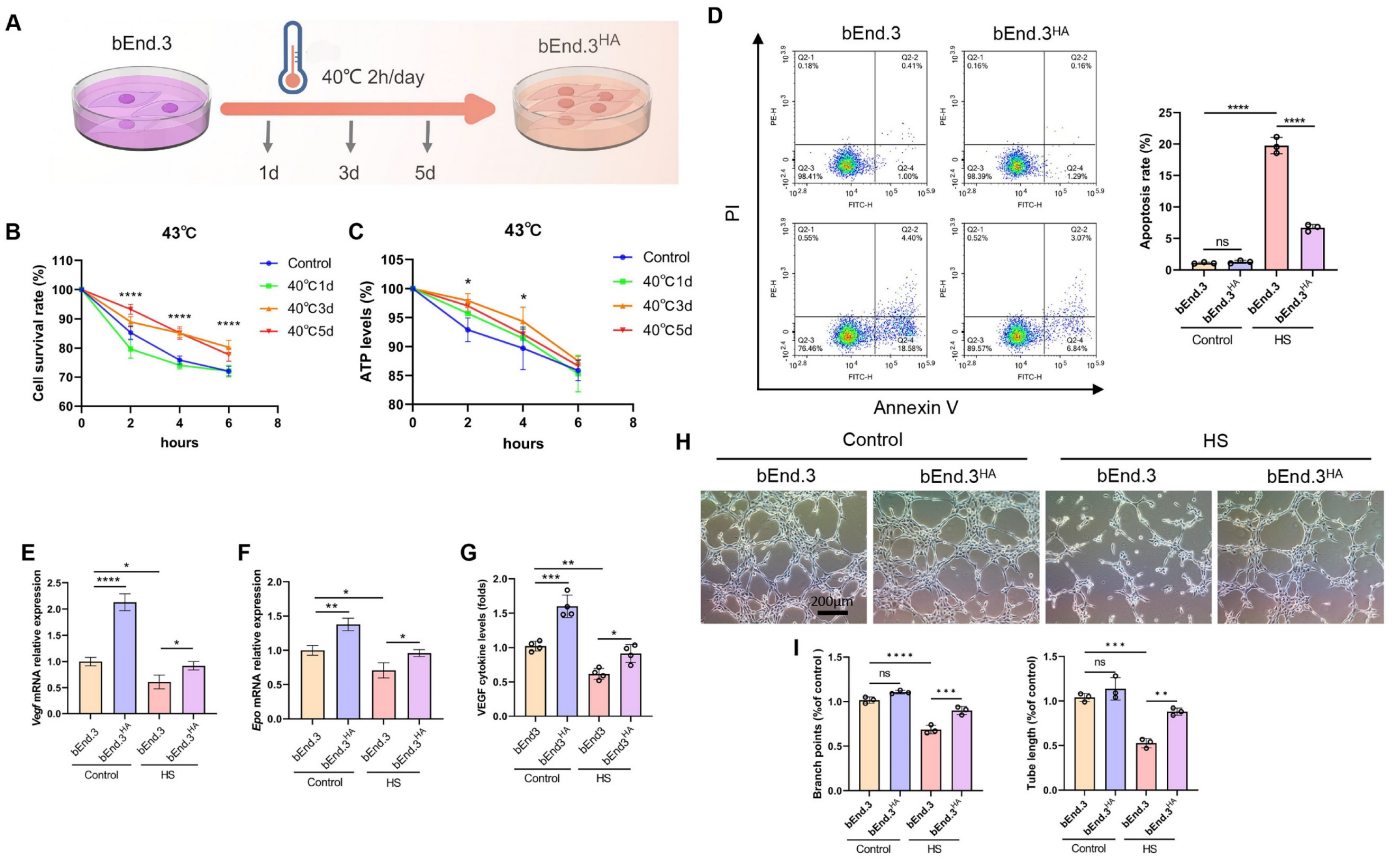
** Intermittent heat stimulation enhances cellular thermotolerance.** A. Schematic diagram of the temperature shift procedure to render bEnd.3 cells into thermoresistant bEnd.3^HA^ (1d, 3d, 5d) cells. **B.** Cell viability of bEnd.3 and bEnd.3^HA^ (1d, 3d, 5d) cells under HS conditions as determined by CCK-8 assay. **C.** ATP levels of bEnd.3 and bEnd.3^HA^ (1d, 3d, 5d) cells under HS conditions as determined by CellTiter-LumiTM assay. **D.** The apoptotic index of bEnd.3 and bEnd.3^HA^ cells under control and HS conditions was determined by flow cytometry. **E & F.** bEnd.3 and bEnd.3^HA^ cells were treated under control and HS conditions. RT-qPCR analyses of vascular endothelial cell markers *Vegf* and *Epo* mRNA. β-actin was used as an internal control. Control: 37℃, HS: 43℃. **G.** ELISA analyses of VEGF protein secretion in culture medium. **H.** Representative images of bEnd.3 and bEnd.3^HA^ on matrigel for 6 h. Scale bars = 200 μm. **I.** Tube length and branch points were quantified. *P < 0.05, **P < 0.01, ***P < 0.001, ****P < 0.0001.

To establish HA cells, bEnd.3 cells were subjected to a temperature scheme to induce thermotolerance. The bEnd.3 cells were exposed to 40℃ for 2 h daily, continuously for 1 day, 3 days, and 5 days (Fig. 1A). When exposed to 43℃ (HS) for 4 h, the bEnd.3 cells exhibited shrinkage, reduced cytoskeleton structures, and decreased ERthermAC fluorescence intensity, along with significantly reduced cell viability and ATP levels, indicating HS sensitivity. In contrast, the bEnd.3 cells subjected to the HA protocol showed significantly improved viability and ATP levels at 43℃, and alleviated cell shrinkage and cytoskeleton reduction (Fig. 1B and 1C; Fig. S1A and S1D). For subsequent experiments, we defined bEnd.3 cells exposed to 40°C cycle for 2 h daily over 3 days as HA cells, and referred to as bEnd.3^HA^ cells in this study. Previous research confirmed that HS significantly enhances cell apoptosis 18. We next examined the impact of HS on apoptosis in bEnd.3 cells. The results showed that, compared to the control group, the apoptosis rate and markers (p53, Bax, and Cleaved-caspase3) were significantly increased under HS in bEnd.3 cells. Conversely, bEnd.3^HA^ cells showed no significant changes in apoptosis rate or the expression of apoptosis genes (Fig. 1D; Fig. S1B and S1C).

To reflect the vascular endothelium health status, we analyzed VEGF, EPO, and VWF expression using qPCR and ELISA. Under HS conditions, bEnd.3 cells had significantly reduced mRNA levels of *Vegf* and *Epo*, and decreased the protein expressions of VWF and VEGF. However, bEnd.3^HA^ cells effectively increased the expression of these markers (Fig. 1E-G, Fig. S1E). Furthermore, under HS conditions, bEnd.3 cells exhibited impaired angiogenesis with fewer branches and shorter tubular structures, while bEnd.3^HA^ cells maintained effective angiogenic ability (Fig. 1H and 1I). The results show that bEnd.3^HA^ cells exhibit significantly improved viability, ATP levels, angiogenic ability, and stable gene expression under HS.

### Intermittent heat stimulation training improves high temperature tolerance in mice

**Figure 2:**
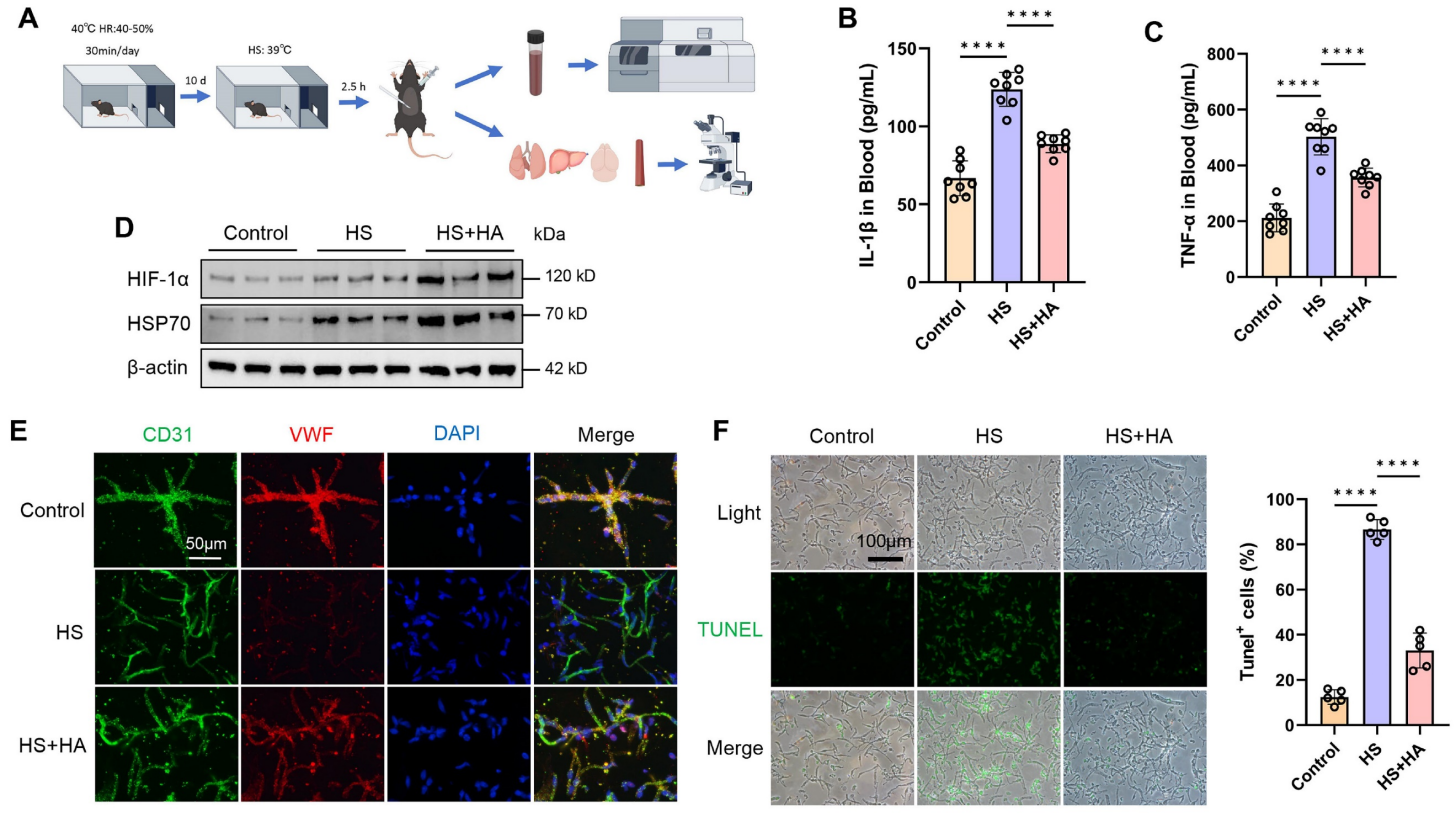
** Intermittent heat stimulation training improves high temperature tolerance in mice. A.** Flowchart of the experiment to improve heat tolerance in mice through intermittent heat stimulation training. **B & C.** ELISA was used to analyze the plasma concentrations of cytokines (IL-1β and TNF-ɑ) in control, HS, and HS+HA group mice (n =8) 16 h after HS. **D.** WB was used to detect the expression of HIF-1α and HSP70 protein. β-actin was used as an internal control. **E.** IF analysis of isolated microvessels stained with antibodies for each cell component. Endothelial markers (CD31, green channel; VWF, red channel) were detected in all microvessel fragments. Scale bar = 50 μm. **F.** IF was used to detect apoptosis in isolated microvessels stained with TUNEL. Scale bar = 100 μm. ****P < 0.0001.

To investigate whether intermittent heat stimulation training can make mice tolerant to high temperatures, the mice were exposed to 40°C for 30 min daily for 10 days. Post-training, both trained and non-trained mice were placed in a high temperature chamber (39°C) for 2.5 h, then housed at room temperature for 16 h before being euthanized for serum and tissue analysis (Fig. 2A). We measured the serum levels of inflammatory factors and found that IL-1β and TNF-α levels increased under HS conditions in mice. However, HA mice had significantly lower levels of IL-1β and TNF-α after HS exposure (Fig. 2B and 2C). Prolonged high temperature exposure causes multi-organ damage 17, 19. Consistent with clinical observations, HS led to neuronal shrinkage in the hippocampal CA3 region, a reduced number of neurons and nissl bodies, hepatocyte necrosis, disorganized alveolar structure, and disordered arrangement of aortic smooth muscle fibers in mice. However, HA mice showed attenuated damage to the brain, liver, lungs, and aorta caused by HS (Fig. S2A and 2B).

To explore HS effects on the brain microvascular endothelium, brain microvessels were isolated using density gradient centrifugation. WB and IF results indicated that, compared to HS mice, HA mice had significantly fewer apoptotic endothelial cells, as well as increased the expression of HIF-1α, HSP70, and VWF (Fig. 2D, 2E, and 2F). These findings suggest that intermittent heat stimulation training in mice reduced inflammatory markers, mitigated multi-organ damage, and enhanced brain microvascular resilience under HS.

### HA induces tolerance to high temperatures via the HIF-1α/HSP70 signaling

**Figure 3:**
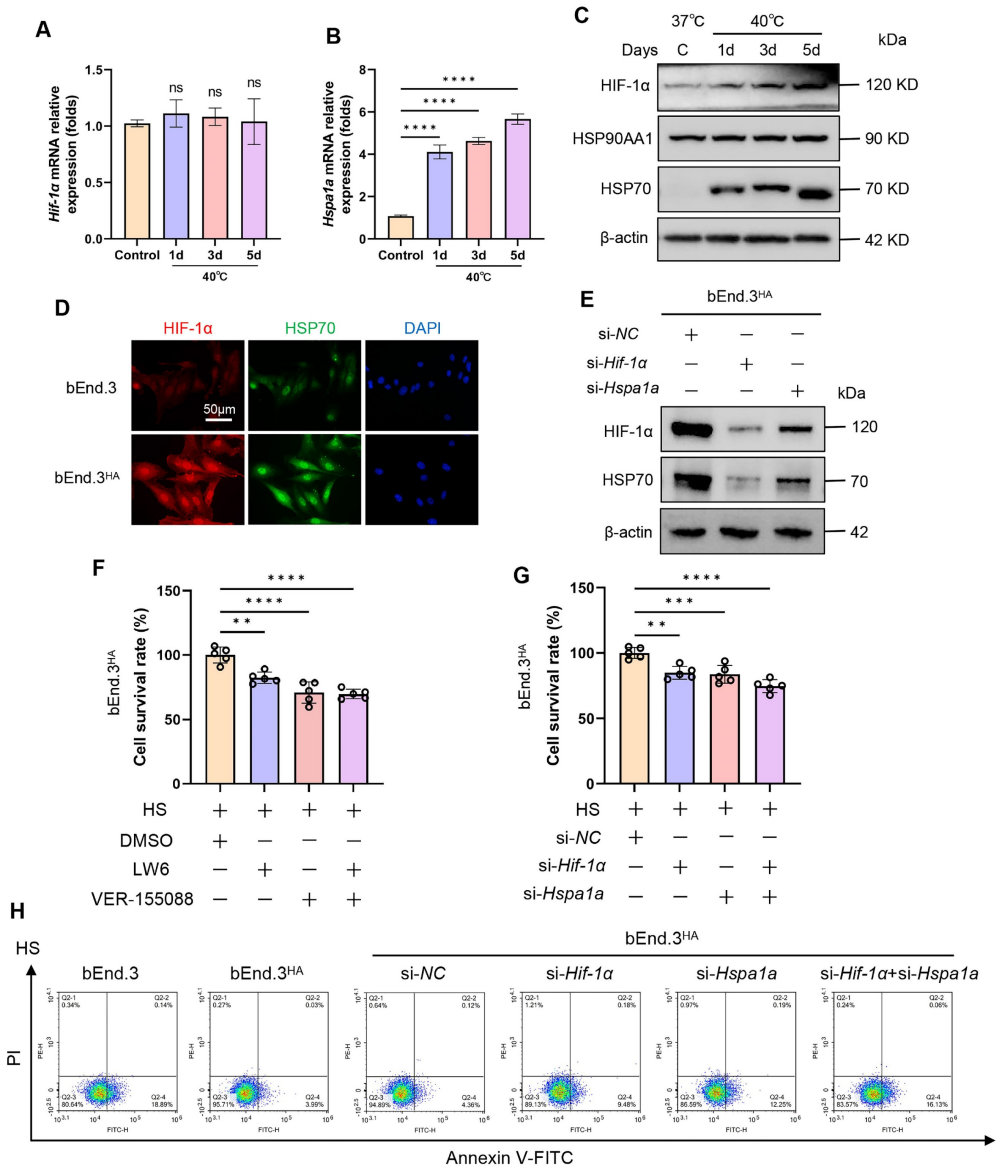
** HA induces tolerance to high temperatures via the HIF-1α/HSP70 signaling. A & B.** The bEnd.3 cells were exposed to 40℃ for 2 h daily, continuously for 1 day, 3 days, and 5 days. RT-qPCR was used to detect the expression of *Hif-1α* and *Hspa1a* mRNA. **C.** WB was used to detect the expression of HIF-1α, HSP90AA1, and HSP70 protein. β-actin was used as an internal control. **D.** IF was used to detect the expression of HIF-1α and HSP70 protein. **E.** WB was used to detect the expression of HIF-1α and HSP70 protein. β-actin was used as an internal control. **F.** bEnd.3^HA^ cells were exposed to HS for 4 h and treated with LW6, VER-155088, or a combination of both. Cell viability was detected using the CCK-8 assay. **G.** bEnd.3^HA^ cells were exposed to HS for 4 h and treated with si-*Hif-1α*, si-*Hspa1a*, or a combination of both. Cell viability was detected using the CCK-8 assay. **H.** Flow cytometry with Annexin V and PI staining was used to determine the apoptotic index of cells. **P < 0.01, ***P < 0.001, ****P < 0.0001.

To explore the molecular mechanisms of HA cells regarding heat tolerance, we first examined the expression of HIF-1α and HSP70 in bEnd.3 cells at different time points (0d, 1d, 3d, 5d) following intermittent heat stimulation. Compared to the control group, both mRNA and protein levels of HSP70 were significantly upregulated, while HIF-1α protein expression markedly increased without a corresponding change in HIF-1α mRNA levels (Fig. 3A-D). Next, we employed inhibitors (LW6, VER-155088) and siRNA (si-*Hif-1α*, si-*Hspa1a*) to suppress HIF-1α and HSP70 expression. The results showed a positive correlation between HIF-1α and HSP70 protein expressions, suggesting a possible mutual regulatory relationship (Fig. 3E; Fig. S3B and 3C). Furthermore, we verified whether the heat tolerance of HA cells depends on the HIF-1α/HSP70 signaling. The results revealed that, compared to control cells, the viability of bEnd.3^HA^ cells was significantly reduced, and cells apoptosis rate was promoted when HIF-1α and HSP70 was silenced by inhibitors or siRNA (Fig. 3F-H). The results show that in bEnd.3^HA^ cells, HIF-1α and HSP70 proteins are upregulated, and inhibiting these proteins reduces cell viability and increases apoptosis, indicating their role in heat tolerance.

### HA enhances HIF-1α protein stability via HSP70 in bEnd.3 cells

**Figure 4:**
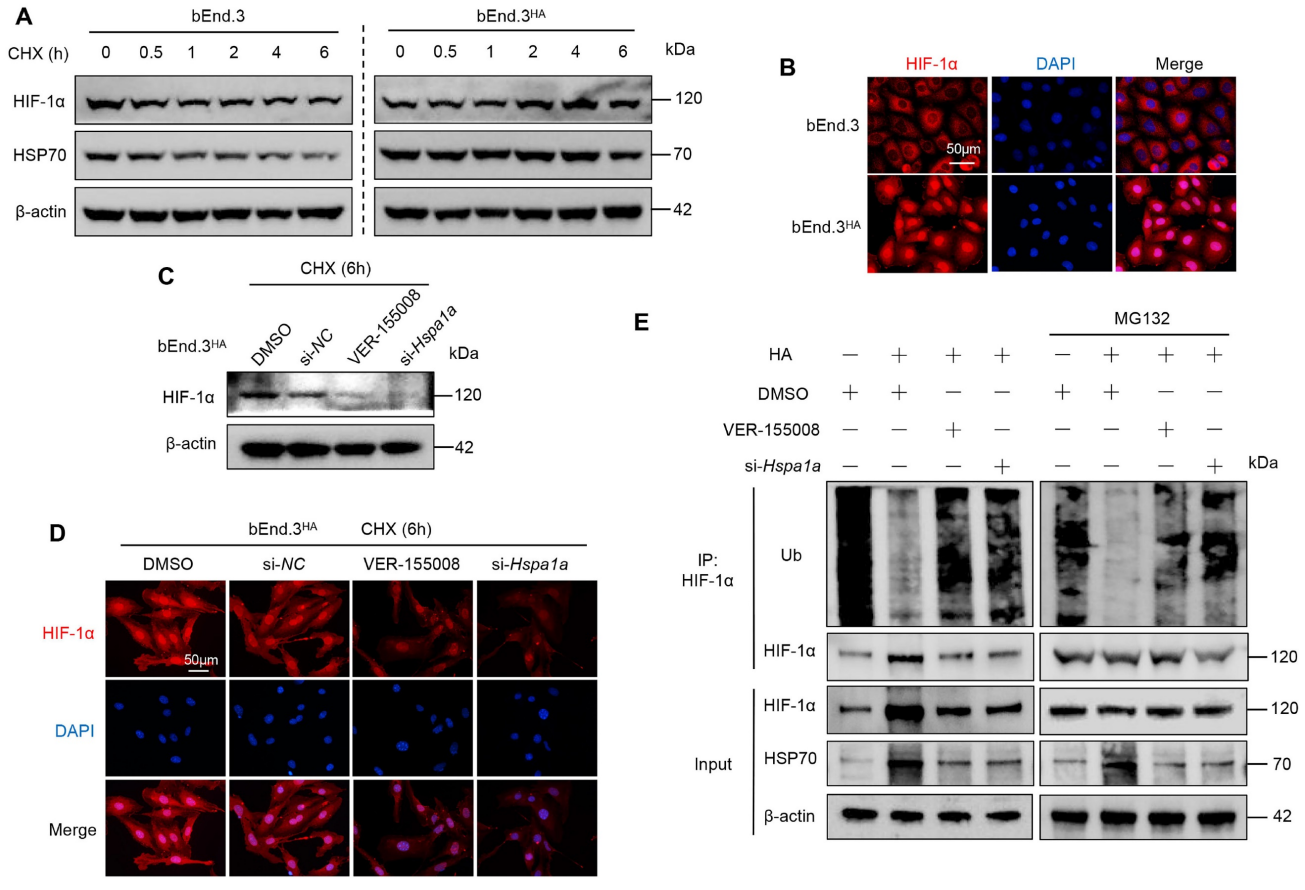
** HA enhances HIF-1α protein stability via HSP70 in bEnd.3 cells. A.** bEnd.3 and bEnd.3^HA^ cells were treated with CHX for the indicated times and analyzed by WB. **B.** IF analysis of HIF-1α subcellular localization. Nuclei are stained with DAPI (blue). n = 5 per group, scar bar = 50 μm. **C.** bEnd.3^HA^ cells were treated with DMSO, si-NC, VER-155008, or si-*Hspa1a* and with CHX for 6 h. WB was used to detect the HIF-1α protein expression. **D.** IF was used to detect the HIF-1α protein expression. Nuclei are stained with DAPI (blue). n=5 per group, scar bar = 50 μm. **E.** bEnd.3 and bEnd.3^HA^ cells were treated with si-NC, VER-155008, or si-*Hspa1a*. The extracts were immunoprecipitated with anti-HIF-1α antibodies and immunoblotted with anti-ubiquitin, anti-HIF-1α, and anti-HSP70 antibodies. β-actin was used as an internal control.

To investigate how HA affects HIF-1α protein stability, we first treated both bEnd.3 and bEnd.3^HA^ cells with CHX for various durations. In bEnd.3 cells, HIF-1α and HSP70 protein levels significantly decreased compared to control cells. However, in bEnd.3^HA^ cells, there were no significant changes in HIF-1α and HSP70 protein levels (Fig. 4A). Notably, HIF-1α localized predominantly in the nucleus of bEnd.3^HA^ cells, whereas in bEnd.3 cells, it was mainly in the cytoplasm (Fig. 4B). These results suggest that HA stabilizes HIF-1α protein and promote its nuclear localization.

To determine whether HSP70 is involved in the effect of HA on HIF-1α protein stability, we treated bEnd.3^HA^ cells with the HSP70 inhibitor (VER-155008) and siRNA (si-*Hspa1a*). Inhibiting HSP70 expression markedly reduced HIF-1α protein levels in CHX-treated bEnd.3^HA^ cells compared to the controls (Fig. 4C and 4D). Next, we further examined the effect of HSP70 on HIF-1α ubiquitination. The CO-IP results showed that HA significantly reduced the ubiquitination level of HIF-1α in bEnd.3 cells, whereas inhibiting HSP70 expression restored the ubiquitination level (Fig. 4E). The results show that in bEnd.3^HA^ cells, HIF-1α and HSP70 protein levels remain stable, while HSP70 inhibition reduces HIF-1α levels and increases its ubiquitination, in contrast to the significant protein level decreases and cytoplasmic localization in bEnd.3 cells.

### HA promotes the interaction between HIF-1α and HSP70 proteins in bEnd.3 cells

**Figure 5:**
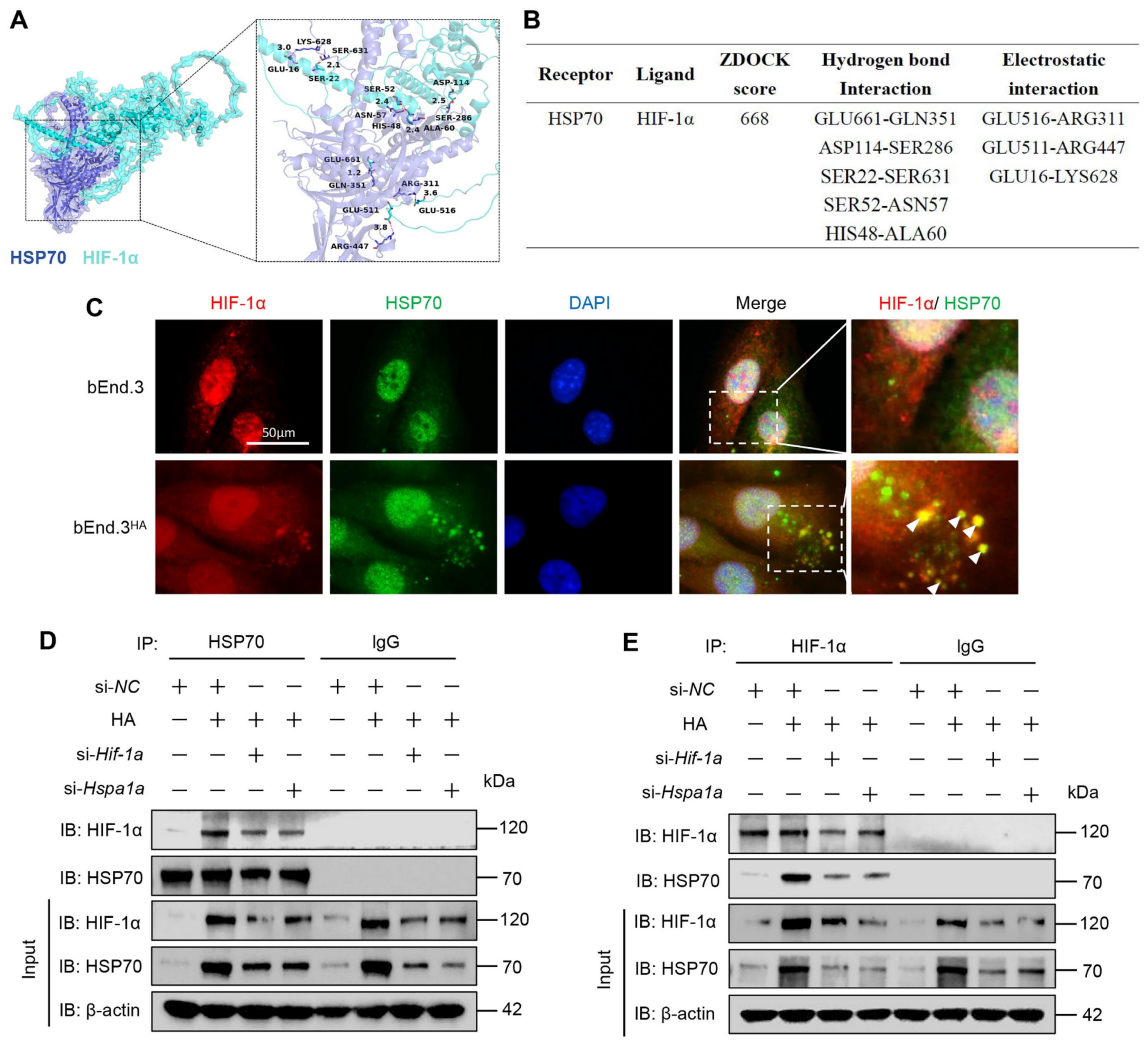
** HA promotes the interaction between HIF-1α and HSP70 proteins in bEnd.3 cells. A.** ZDOCK prediction was used to model the predicted interaction between HSP70 and HIF-1α. **B.** Predicted ZDOCK binding score, hydrogen bonds, and electrostatic interactions with amino acid sites between HSP70 and HIF-1α. **C.** Colocalization of HIF-1α (red) with HSP70 (green) in bEnd.3 and bEnd.3HA cells. Nuclei are stained with DAPI (blue). n=5 per group, scar bar = 50 μm. **D & E.** bEnd.3 and bEnd.3HA cells were treated with si-NC, si-Hif-1α, or si-Hspa1a. The extracts were immunoprecipitated with anti-HIF-1α or anti-HSP70 antibodies and immunoblotted with anti-HIF-1α and anti-HSP70 antibodies.

To assess the binding affinity between HSP70 and HIF-1α proteins, we conducted a molecular docking study. The ZDOCK Score values and their best pose interaction were calculated (Fig. 5A). The ZDOCK Score of HSP70 and HIF-1α was 668. As shown in Fig. 5B, HSP70 forms hydrogen bond links with amino acid sites such as GLU661-GLN351, ASP114-SER286. Comprehensive analysis revealed that proteins HSP70 and HIF-1α formed a stable locking model. This predicted interaction was validated through colocalization and CO-IP experiments. IF experiment results showed significant colocalization of HIF-1α and HSP70 in the cytoplasm of bEnd.3^HA^ cells compared to bEnd.3 cells (Fig. 5C). Subsequently, using antibodies against HIF-1α or HSP70 to pull down cell lysates, we confirmed that HA promotes the interaction between endogenous HIF-1α and HSP70 proteins (Fig. 5D and 5E). These results indicate that HA enhances interaction between HIF-1α and HSP70 proteins and significant cytoplasmic colocalization in bEnd.3^HA^ cells.

### HA induces tolerance to hypoxia via the HIF-1α/HSP70 signaling

**Figure 6:**
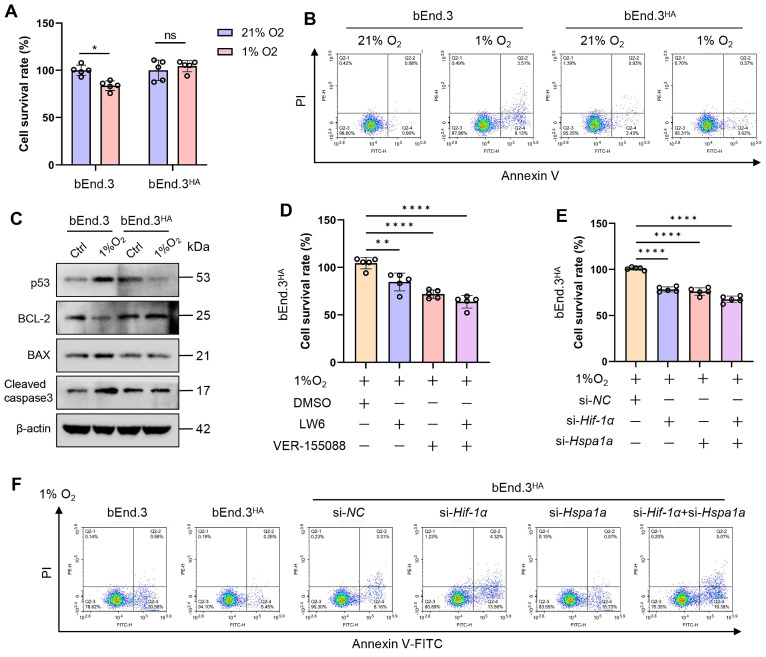
** HA induces tolerance to hypoxia via the HIF-1α/HSP70 signaling. A.** bEnd.3 and bEnd.3^HA^ cells were treated at 1% O_2_ concentration for 12 h. Cell viability was detected using the CCK-8 assay. **B.** Flow cytometry with Annexin V and PI staining was used to determine the apoptotic index of cells. **C.** WB was used to detect the expression of p53, Bcl-2, Bax, and cleaved caspase3 protein. β-actin was used as an internal control. **D.** bEnd.3^HA^ cells were treated at 1% O_2_ concentration for 12 h and with LW6, VER-155008, or LW6+VER-155008. Cell viability was detected using the CCK-8 assay. **E.** bEnd.3^HA^ cells were treated at 1% O_2_ concentration for 12 h and with si-*Hif-1α*, si-*Hspa1a*, or si-*Hif-1α*+si-*Hspa1a*. Cell viability was detected using the CCK-8 assay. **F.** Flow cytometry with Annexin V and PI staining was used to determine the apoptotic index of cells. *P < 0.05, **P < 0.01, ***P < 0.001.

To explore the molecular mechanisms underlying the tolerance of HA cells to hypoxia, we first cultured bEnd.3 cells in an incubator with 1% O₂ for various time points. The results showed that as the hypoxia treatment period extended, cell viability decreased, and apoptosis rate significantly increased. Additionally, the levels of apoptosis molecules, p53 and Bax proteins, were upregulated, while Bcl-2 protein levels were downregulated (Fig. S4A-C). Given that previous studies indicating hypoxia promotes HIF-1α and HSP70 expression 20, 21, we assessed the protein expression of HIF-1α, HSP70, and HSP90AA1 during hypoxia. The results showed that hypoxia significantly upregulated HIF-1α and HSP70 protein levels, but not HSP90AA1, compared to control group (Fig. S4D). We next verified whether bEnd.3^HA^ cells exhibit tolerance to hypoxia. Experimental results revealed no significant differences in cell viability, apoptosis rate, and the expression of apoptosis proteins (p53, Bcl-2, Bax, cleaved caspase-3) in bEnd.3^HA^ cells under hypoxic conditions compared to the control group (21% O₂) (Fig. 6A-C). Subsequently, Inhibiting HIF-1α and HSP70 with inhibitors and siRNA under hypoxic conditions significantly reduced bEnd.3^HA^ cell viability and induced apoptosis compared to control group (Fig. 6E-G). The findings indicate that, under hypoxic conditions, bEnd.3^HA^ cells maintain cell viability and apoptosis rate compared to bEnd.3 cells, while inhibiting HIF-1α and HSP70 results in decreased viability and induced apoptosis.

### Intermittent heat stimulation training in mice to hypoxia tolerance

**Figure 7:**
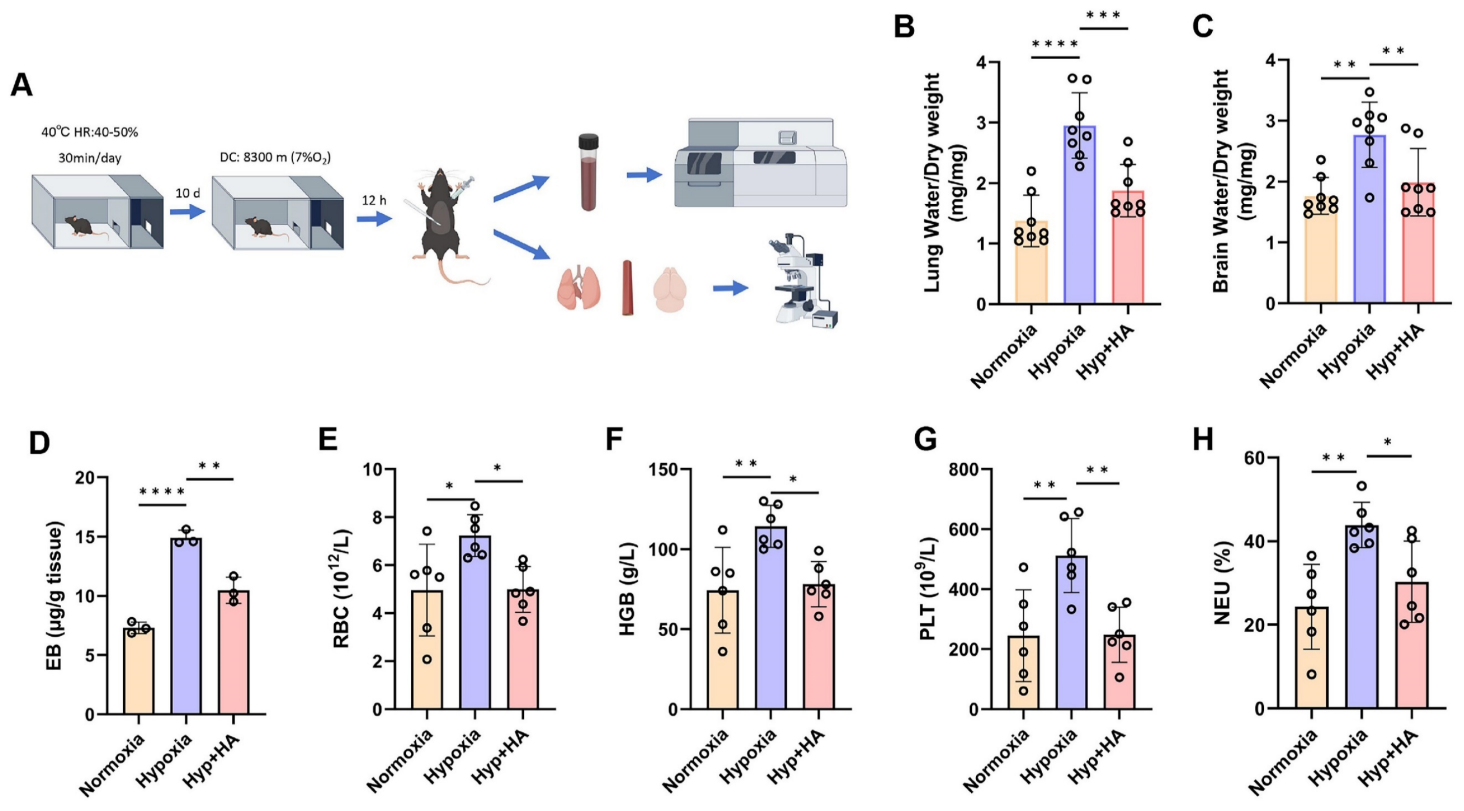
** Intermittent heat stimulation training improves hypoxia tolerance in mice. A.** Flowchart of the experiment to improve hypobaric hypoxia tolerance in mice through intermittent heat stimulation training. **B & C.** The water content in the brain and lung of mice (n = 8). **D.** The EB extravasation assay was used to evaluate the BBB permeability (n = 3). **E-H.** The levels of red blood cells (RBC), hemoglobin (HGB), platelet (PLT), and neutrophil (NEU) in mice (n = 6). *P < 0.05, **P < 0.01, ***P < 0.001, ****P < 0.0001.

To determine whether intermittent heat stimulation training enhances hypoxia tolerance, both control and HA mice were placed in a hypobaric hypoxia chamber (8300 m, 7% O₂) for 12 h before euthanasia. Blood and tissue samples were then collected for analysis (Fig. 7A). Considering the sensitivity of the central nervous system and lungs to acute hypoxia, as well as the role of tissue edema in hypoxia 22, we assessed whether HA reduces hypoxic cerebral and pulmonary edema. The results showed that after hypobaric hypoxia exposure, control mice exhibited mild cerebral and pulmonary edema, whereas HA mice had significantly less edema (Fig. 7B and 7C). Cerebral edema, associated with increased BBB permeability 23, was assessed using the EB dye method. Under hypobaric hypoxia, control mice showed a significant increase in EB concentration in brain tissues, indicating higher BBB permeability. HA mice had significantly lower EB concentration, suggesting that HA protects against hypoxia-induced permeability increases (Fig. 7D). Complete blood count results after hypoxia showed significant increases in red blood cells, hemoglobin levels, platelet counts, and neutrophil percentages in control mice, but not in HA mice (Fig. 7E-H). Additionally, HA partially mitigated damage to the brain, lungs, and aorta caused by hypobaric hypoxia (Fig. S5A). These results show that under hypoxic conditions, HA mice exhibited significantly less cerebral and pulmonary edema, lower concentrations of brain EB, stable blood and biochemical parameters, and reduced organ damage compared to control mice.

## Discussion

HA refers to the process by which organisms adapt to high temperature environments through a series of physiological and biochemical reactions following short-term exposure. This adaptation can enhance the ability of body to cope with other extreme conditions. However, the exact molecular mechanisms underlying the role of HA in extreme environment tolerance remain unclear. Our research indicates that HA enhances the interaction between HIF-1α and HSP70 proteins, stabilizing the HIF-1α protein and contributing to tolerance to both heat and hypoxia. This study reveals that the cell protection mechanisms mediated by HA provide new strategies and potential targets for human adaptation to extreme environments.

Prolonged exposure to extreme environments, including high temperatures, low oxygen, high radiation, and high-pressure conditions, can compromise the integrity and function of the BBB, increasing the risk of central nervous system disorders. Research indicates that protecting and repairing the BBB is crucial for individuals living and working in such environments 24-26. Our study found that in high temperature and low-oxygen environments, the BBB in mice was significantly damaged, resulting in increased permeability. However, HA mice effectively alleviated brain damage caused by these conditions. Brain microvascular endothelial cells, which are the main components of the BBB, play a critical role in resisting various environmental stress and maintaining central nervous system homeostasis 27, 28. Therefore, we used brain microvascular endothelial cells as the *in vitro* cell study model. In this study, we discovered that intermittent heat stimulation enhanced the tolerance of brain microvascular endothelial cells and mice to high temperatures, and improved the tolerance of mice to extreme low-oxygen environments while protecting BBB function. Our research introduces a novel, efficient, and safer adaptive training method for human programs. Practically, similar intermittent heat stimulation protocols can be developed and tailored to individual physiological parameters such as age, gender, health, fitness, and adaptability. These protocols involve training in a controlled environment with repeated exposure to moderate high temperatures, followed by sufficient recovery time to normal temperature, enhancing blood-brain barrier protection. This method can be integrated into the training of athletes, military personnel, and workers in extreme environments to boost adaptability and performance while minimizing central nervous system health risks.

In the process of HA, the key regulatory factors HIF-1α and HSP70 play a central role, independently regulating the cellular response to HS and working synergistically to enhance organism tolerance and adaptability 29, 30. Previous studies have shown that post-HA, the levels of HIF-1α and HSP70 in cardiac tissue significantly increase, providing additional protection to the heart 13. This indicates that cells establish a multi-layered defense mechanism by utilizing existing HIF-1α and HSP70 proteins and rapidly activating an emergency alert system, thereby enhancing resistance to various stressors 13, 30. Although HIF-1α and HSP70 play crucial roles in mediating tolerance to other stress environments during HA, the specifics of their upstream and downstream regulatory relationships and synergistic mechanisms remain unclear. Consistent with previous studies, the expression of HIF-1α and HSP70 proteins was significantly upregulated in brain microvascular endothelial cells during HA. Inhibiting HIF-1α or HSP70 expression partially reduced the tolerance to high temperatures and hypoxia, suggesting that the tolerance effects of HA to high temperatures and hypoxia depend on the HIF-1α/HSP70 signaling.

HIF-1α is crucial for sensing hypoxia. Under normal conditions, the HIF-1α protein is rapidly degraded post-translation. However, under hypoxic conditions, the ubiquitination degradation pathway of HIF-1α is inhibited, leading to protein accumulation 31. Additionally, HIF-1α functions as a transcription factor, regulating the expression of over 40 downstream genes 31, thereby playing an important role in cellular stress tolerance. Recent research indicates that the sustained transcriptional activation of HIF-1α is essential for the cross-tolerance mediated by HA, highlighting its importance in hypoxia tolerance facilitated by HA 32. Another study found that HSP90 can interact with HIF-1α under hypoxic conditions, increasing its stability 33. In our study, HA was found to reduce the ubiquitination level of HIF-1α protein, while inhibition of HSP70 restored HIF-1α ubiquitination. Interestingly, HA promoted the interaction between HIF-1α and HSP70 proteins, suggesting that HA may enhance the interaction between HIF-1α and HSP70, reduce HIF-1α protein ubiquitination, and thereby increase its stability. Although our findings further elucidate the molecular mechanisms of HA-mediated cellular protection, further research is needed to uncover deeper molecular mechanisms. Specifically, a detailed investigation into the specific binding sites, spatial binding modes, and other potential molecules involved in the interaction of HSP70 and HIF-1α is necessary. Additionally, validating the generalizability and applicability of these findings in animal models and clinical human studies is essential. This would provide stronger scientific evidence for understanding and applying HA training strategies to combat extreme environments.

**Figure 8:**
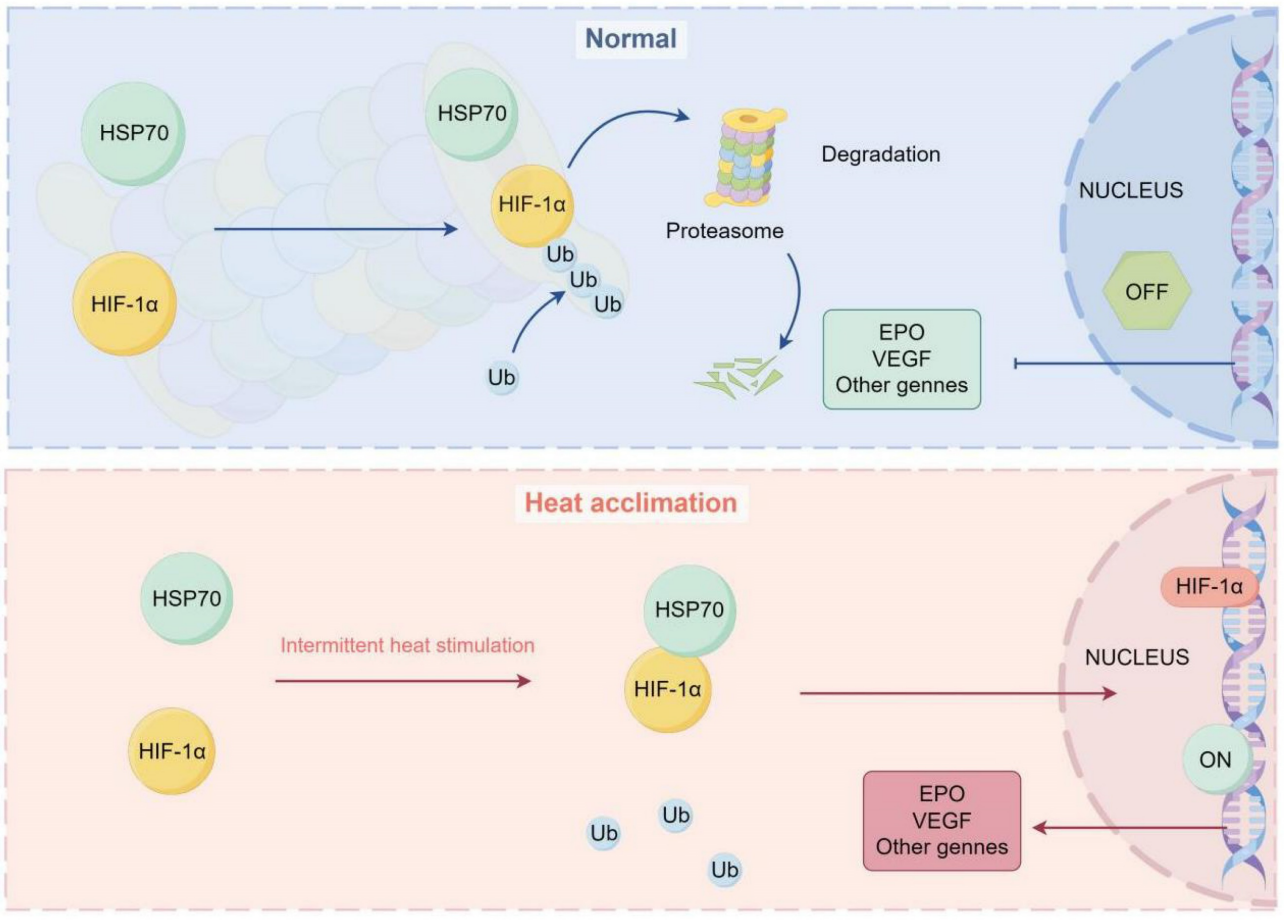
** Diagram representing the potential mechanism by which HA regulates stabilization of HIF-1α protein via HSP70 in bEnd.3 cells.** Under normal conditions, HIF-1α protein is ubiquitinated and degraded by the proteasome. Under HA conditions, HSP70 interacts with the HIF-1α protein, inhibiting its ubiquitination, stabilizing the HIF-1α protein, and promoting the transcription of downstream genes in the nucleus.

In summary, our results reveal that HA reduces the ubiquitination level of HIF-1α protein in brain microvascular endothelial cells by enhancing the interaction between HSP70 and HIF-1α proteins, thereby increasing HIF-1α stability (Fig. 8). Our findings further clarify the potential molecular mechanisms of HA-mediated cellular protection, providing new strategies and potential targets for human adaptation to extreme environments.

## Supplementary Material

Supplementary methods, figures and tables.

## Abbreviations

HA: heat acclimation
HS: heat stress
HIF-1α: hypoxia-inducible factor 1-alpha
HSP70: heat shock protein 70
HPC: hippocampus
EB: evans blue
BBB: blood-brain barrier permeability
RBC: red blood cells
HGB: hemoglobin
PLT: platelet
NEU: neutrophil
HE: hematoxylin and eosin
CCK-8: cell counting kit-8
DMEM: dulbecco's modified eagle medium
FBS: fetal bovine serum
P/S: penicillin/streptomycin
IF: immunofluorescence
WB: western blot
qPCR: quantitative polymerase chain reaction
CO-IP: co-immunoprecipitation
CHX: cycloheximide
