# Supplementary material for: Heat acclimation mediates cellular protection via HSP70 stabilization of HIF-1α protein in extreme environments
Source: Int J Biol Sci. 2025 Jan 1;21(1):175–88. doi: 10.7150/ijbs.103122 (PMC11667810; doi:10.7150/ijbs.103122)
Supplement: Supplementary file 1 — Supplementary methods, figures and tables. [file ijbsv21p0175s1.pdf]

Supplementary Materials

Heat acclimation mediates cellular protection via HSP70 stabilization of HIF-1α protein in extreme environments

Materials and Methods

Cell HS experiment

Once 80-90% confluency was reached, bEnd.3 cells were passaged and seeded into 6 cm culture dishes. The cells were incubated at 37°C with 5% CO_2 until the cell density reached approximately 70%, typically by the next day. Subsequently, the cells were exposed to a 43°C incubator with 5% CO_2 for intervals of 0, 2, 4, 6, 9, and 12 h, respectively. Following incubation, collect the cells, extract total protein and RNA, and store the samples at -80°C.

Biotracker ERthermAC staining

The bEnd.3 cells were cultured in incubators at 37℃and 43℃ for 4 h. ERthermAC was then added to the culture medium to reach a final concentration of 250 nM, and the incubation was continued at 37 ℃ for an additional 30 min. Subsequently observed under a fluorescence microscope.

IF staining

The bEnd.3 and bEnd.3^{HA} cells were digested, centrifuged, and resuspended. Subsequently, the cells were seeded into a 24-well plate at a density of $2\times10^{\wedge 4}$ cells per well. The plates were then incubated at 43°C with 5% CO_2 for 4 h. Following incubation, the cells were fixed with 10% neutral formaldehyde. Permeabilization was performed using 0.3% Triton X-100, followed by blocking with 10% goat serum. Vari fluor 488-phalloidin were added and incubated for 20 min at RT. The cells were washed with PBS, incubated with DAPI, mounted with glycerol, and subsequently observed under a fluorescence microscope.

The cells treatment method was performed as described above. Following incubation, the cells were fixed with 10% neutral formaldehyde. Permeabilization was performed using 0.3% Triton X-100, followed by blocking with 10% goat serum. Primary antibodies anti-VWF were added and incubated overnight at 4°C in the dark. The cells were washed with PBS, fluorescent

secondary antibodies were added and incubated for 2 h at 37°C, incubated with DAPI, mounted with glycerol, and subsequently observed under a fluorescence microscope.

Histological analysis

After the experiment ended, the mice were euthanized, and the entire brains, livers, lungs, and aortas were collected. These tissues were fixed in 4% paraformaldehyde, dehydrated, and embedded. Continuous sections 4 micrometers thick were prepared using paraffin slicing techniques. The tissue sections were then stained with Hematoxylin and Eosin (HE) and Nissl stain according to the protocol. Finally, photographs were taken and analyzed under an optical microscope.

Cell viability

The bEnd.3 cells were digested, centrifuged, and resuspended. Subsequently, the cells were seeded into a 96-well plate at a density of $1\times10^{\wedge4}$ cells per well. The plates were then incubated at 37°C with 1% O_2 for 12, 24, and 48 h respectively. Thereafter, CCK-8 solution was added, and the plates were incubated at 37°C for 1 h. Finally, absorbance was measured at 450 nm using a microplate reader.

Cell apoptosis

The bEnd.3 cells were digested, centrifuged, and resuspended. Subsequently, the cells were seeded into 6 cm culture dishes. The cells were incubated at 37°C with 1% O_2 for 12, 24, and 48 h respectively. Next, the cells were collected and each group was processed according to the reagent instructions. Annexin V-FITC and PI reagents were then added, and the samples were incubated in the dark at room temperature for 15 minutes. The apoptosis rate was detected using a flow cytometer.

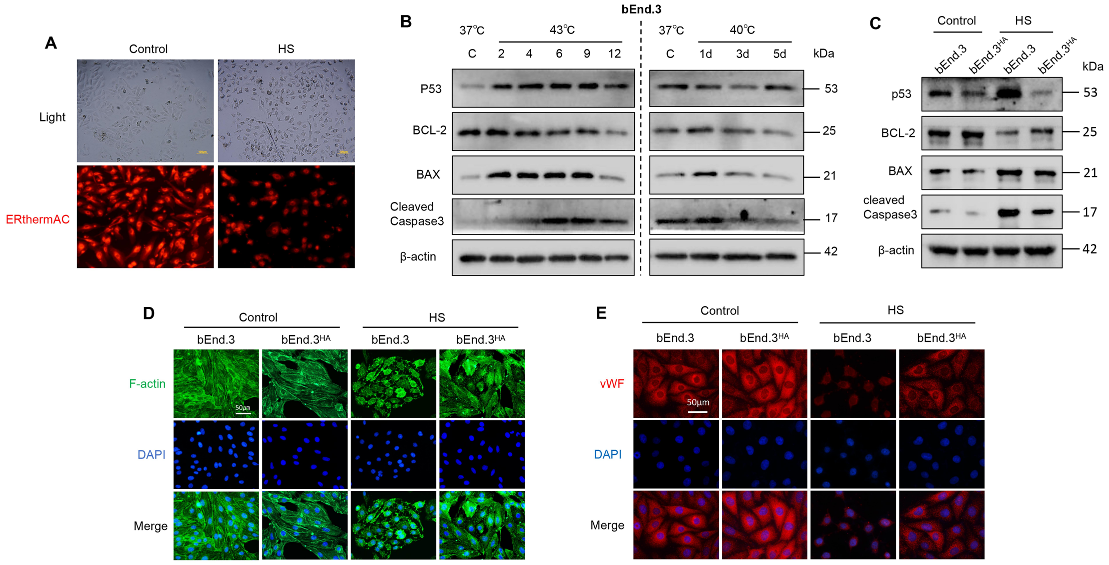

Supplemental Figure 1. Intermittent heat stimulation enhances cellular thermotolerance. A. The ERthermAC (red) fluorescence intensity of bEnd.3 cells under control and HS conditions was determined by fluorescence microscope. **B.** p53, Bax, Bcl-2, and cleaved Caspase3 protein levels of bEnd.3 cells under different temperature conditions was determined by western blot. **C.** p53, Bax, Bcl-2 and cleaved Caspase3 protein levels of bEnd.3 and bEnd.3^{HA} cells under control and HS conditions was determined by WB. β-Actin was used as the internal control. Control: 37°C, HS: 43°C. D. F-actin expression of bEnd.3 and bEnd.3^{HA} cells under control and HS conditions was determined by IF staining. E. IF staining analyses of VWF protein. n = 3 independent biological repeats. *$P < 0.05$, ****$P < 0.0001$.

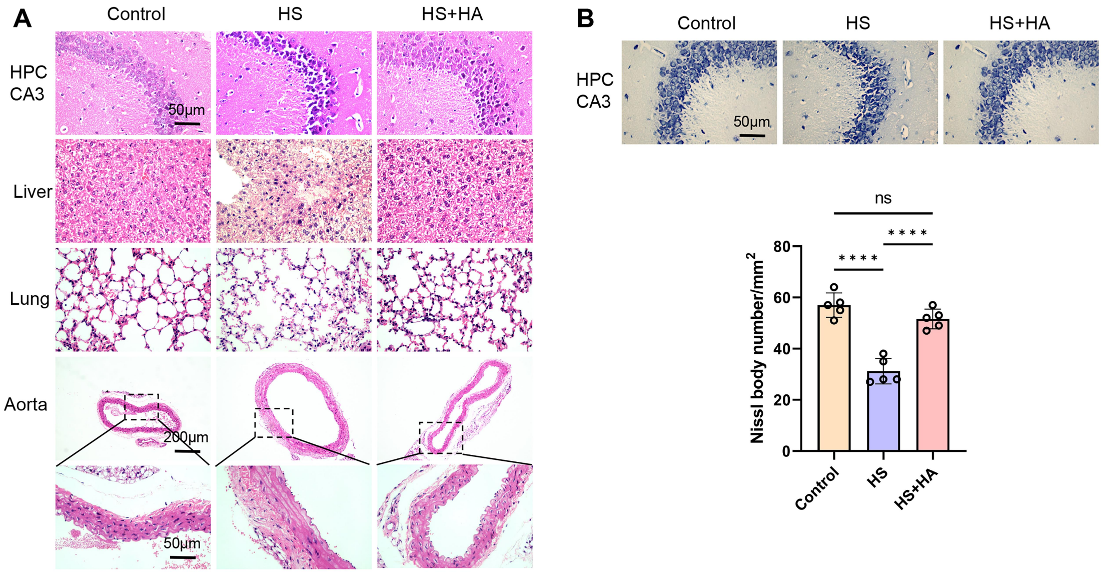

Supplemental Figure 2. Intermittent HS training improves high temperature tolerance in mice. A. Representative images of HE staining of different organs from Control, HS, or HS+HA group mice (n = 8) 16 h after HS. Scale bar = 50 μm. **B.** Representative images of nissl staining of hippocampal from Control, HS, or HS+HA group mice (n = 8) 16 h after HS. Scale bar = 50 μm. ****$P < 0.0001$.

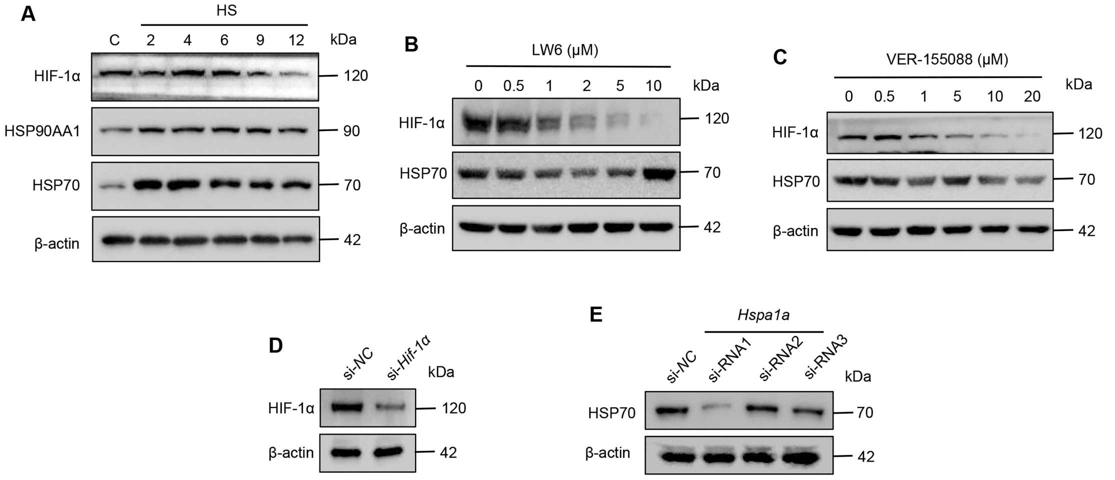

Supplemental Figure 3. HA induces tolerance to high temperatures via the HIF-1α/HSP70 signaling. A. bEnd.3 cells were exposed to HS for indicated times (0, 2, 4, 6, 9, 12 h). WB was used to detect the expression of HIF-1α, HSP90AA1, and HSP70 protein. **B.** bEnd.3 cells were treated with LW6 at the indicated concentrations (0, 0.5, 1, 2, 5, 10 μM) for 24 h. WB was used to detect the expression of HIF-1α and HSP70 protein. **C.** bEnd.3 cells were treated with VER-155088 at the indicated concentrations (0, 0.5, 1, 5, 10, 20 μM) for 24 h. WB was used to detect the expression of HIF-1α and HSP70 protein. **D.** bEnd.3 cells were treated with si-*NC* and si-*Hif-1α*. WB was used to detect the expression of HIF-1α. **E.** bEnd.3 cells were treated with si-*NC* and si-*Hspa1a* (si-RNA1, si-RNA2, si-RNA3). WB was used to detect the expression of HSP70. β-actin was used as an internal control. n = 3 independent biological repeats.

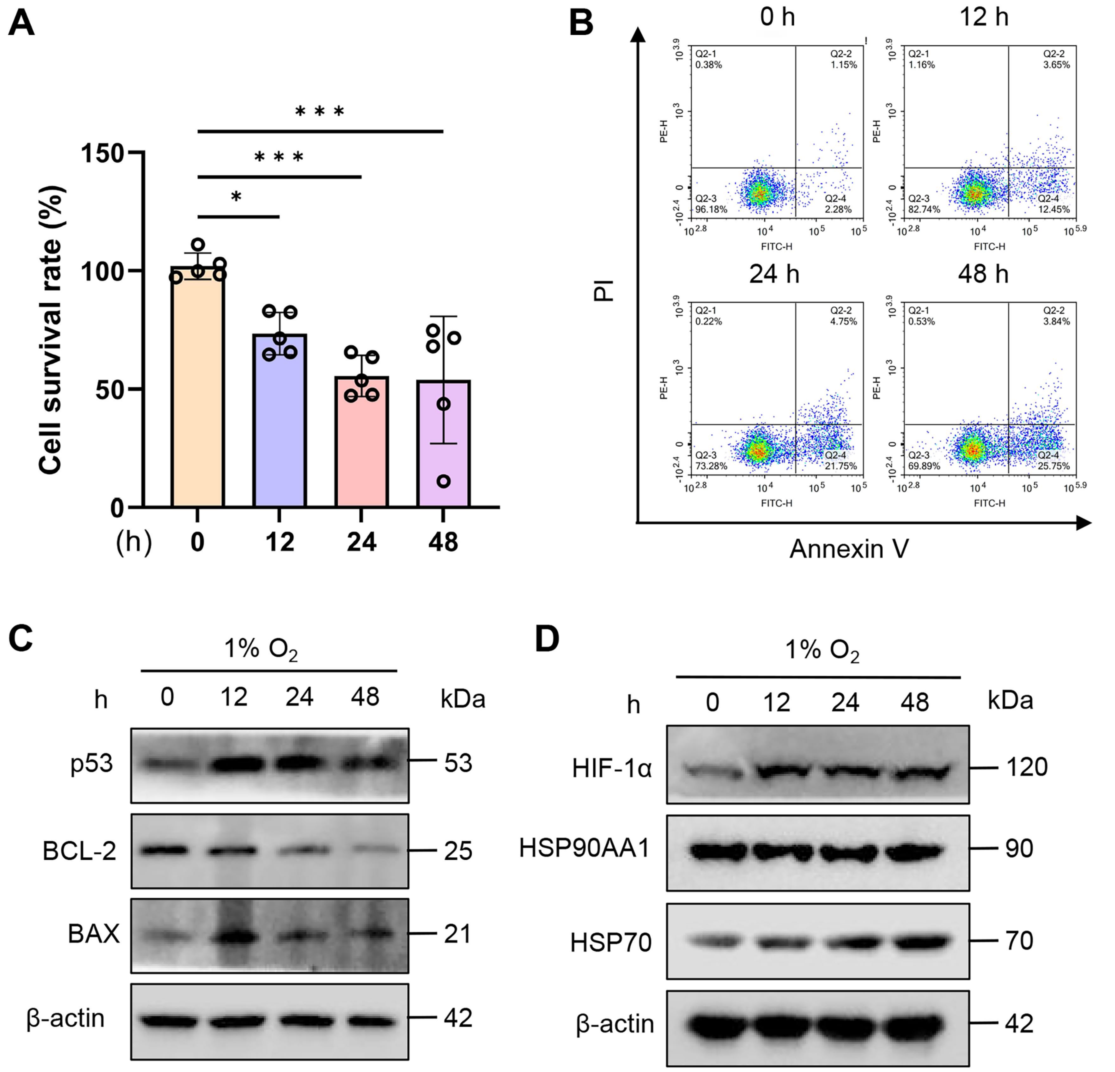

Supplemental Figure 4. Hypoxia induces apoptosis in bEnd.3 cells. A. bEnd.3 cells were treated at 1% O_2 concentration for varying durations (0, 12, 24, 48 h). Cell viability was detected using the CCK-8 assay. **B.** Flow cytometry with Annexin V and PI staining was used to determine the apoptotic index of cells. **C & D.** WB was used to detect the expression of p53, Bcl-2, Bax, HIF-1α, HSP90AA1, and HSP70 protein. β-actin was used as an internal control. n = 3 independent biological repeats. *$P < 0.05$, ***$P < 0.001$.

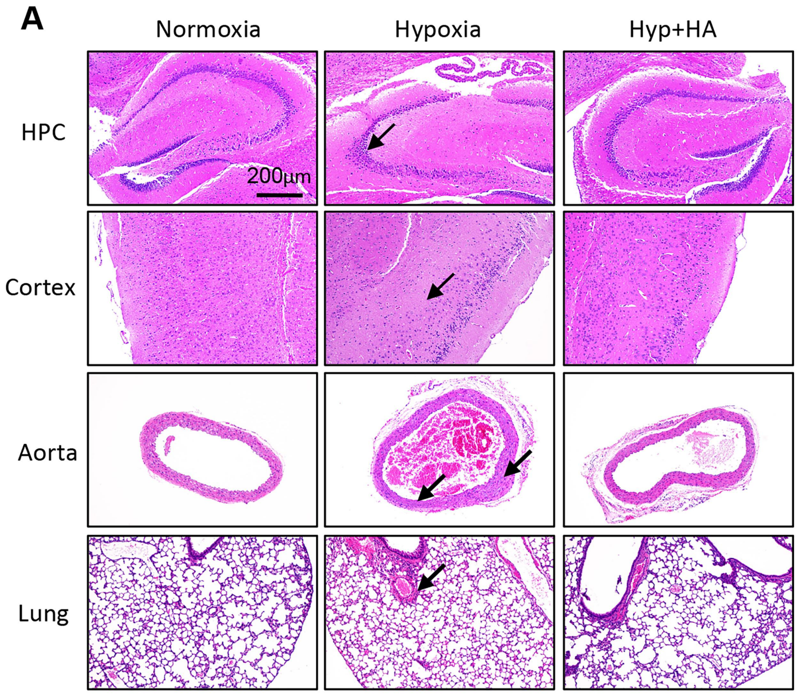

Supplemental Figure 5. Intermittent heat stimulation training improves hypoxia tolerance in mice. A. Representative HE stains in the hippocampus (HPC) and cortex of the mice brain, aortic blood vessels, and lung tissue (n = 8). Scale bar = 200μm.

Table S1. The siRNA sequences were as follows:

Name	siRNA sequence
si-m-*Hspa1a*-1	5′-GCGUGUUCCAGCACGGCAATT-3′ 5′-UUGCCGUGCUGGAACACGCTT-3′
si-m-*Hspa1a*-2	5′-CGCAGACCUUCACCACCUATT-3′ 5′-UAGGUGGUGAAGGUCUGCGTT-3′
si-m-*Hspa1a*-3	5′-GCGAGGCUGACAAGAAGAATT-3′ 5′-UUCUUCUUGUCAGCCUCGCTT-3′
si-m-*Hif-1α*	5′-GAUGUGACAUCCACAGACGAA-3′ 5′-UUCGUCUUGGGAUGUCACUAG-3′
si-*NC*	5′-UUCUCCGAACGUGUCACGUTT-3′ 5′-ACGUGACACGUUCGGAGAATT-3′

Table S2. Primer pairs used for RT-qPCR

Gene	Sequence
Mouse *Vegf*	F 5′-GCACATAGAGAGAATGAGCTTCC-3′<br>R 5′-CTCCGCTCTGAACAAGGCT-3′
Mouse *Epo*	F 5′-ACTCTCCTTGCTACTGATTCCT-3′<br>R 5′-ATCGTGACATTTTCTGCCTCC-3′
Mouse *Hspa1a*	F 5′-GAGATCGACTCTCTGTTCGAGG-3′<br>R 5′-GCCCGTTGAAGAAGTCCTG-3′
Mouse *Hif-1a*	Fw 5′-ACCTTCATCGGAAACTCCAAAG-3′

	Rv 5′-CTGTTAGGCTGGGAAAAGTTAGG-3′
Mouse *Actb*	F 5′-CCACCATGTACCCAGGCATT-3′
	R 5′-CAGCTCAGTAACAGTCCGCC-3′

Abbreviations: Fw, forward primer; RT-qPCR, reverse-transcription quantitative polymerase chain reaction; Rv, reverse primer.
